# Supplementary material for: Screening for Hyperthermophilic Electrotrophs for the Microbial Electrosynthesis of Organic Compounds
Source: Microorganisms. 2022 Nov 14;10(11):2249. doi: 10.3390/microorganisms10112249 (PMC9696306; doi:10.3390/microorganisms10112249)
Supplement: Supplementary file 1 [file microorganisms-10-02249-s001.zip › microorganisms-2014865-supplementary.pdf]

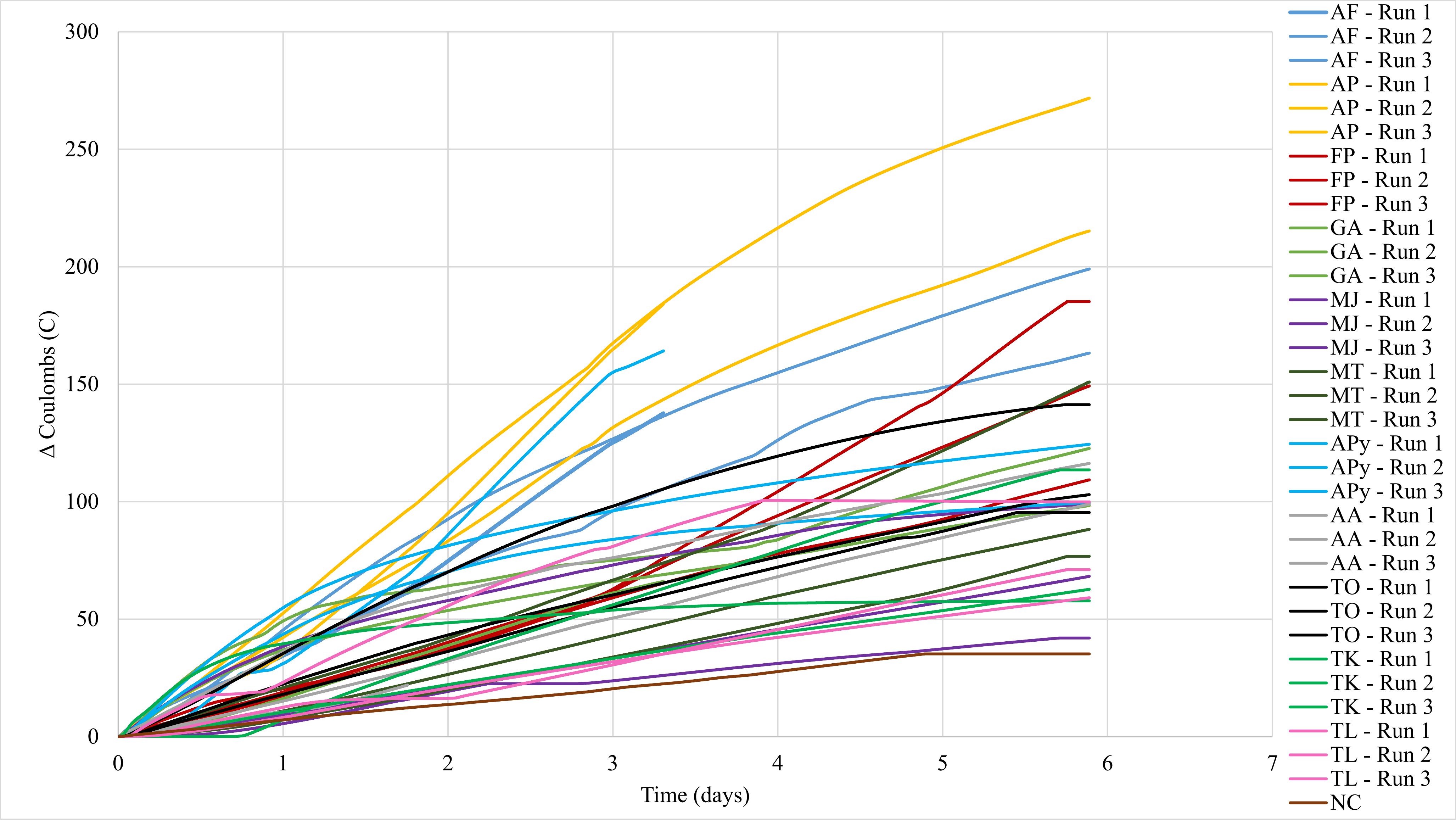

Figure S1. Coulombs consumed over time during the triplicates of the screening of the 11 (hyper)thermophilic strains. Data are missing from day 3.3 on the first run of AF, AP, GA, Apy, and AA due to technical problems and were extrapolated for further analysis. AF: *Archaeoglobus fulgidus;* AP: *Archaeoglobus profundus*; FP: *Ferroglobus placidus*; GA: *Geoglobus ahangari*; MJ: *Methanocaldococcus jannaschii*; MT: *Methanococcus thermolithotrophicus*; APy: *Aquifex pyrophilus*; AA: *Aquifex aeolicus*; TO: *Thermococcus onnurineus*; TK: *Thermococcus kodakarensis*; TL: *Thermococcus litoralis*. NC: negative control with mix of electron acceptor used.

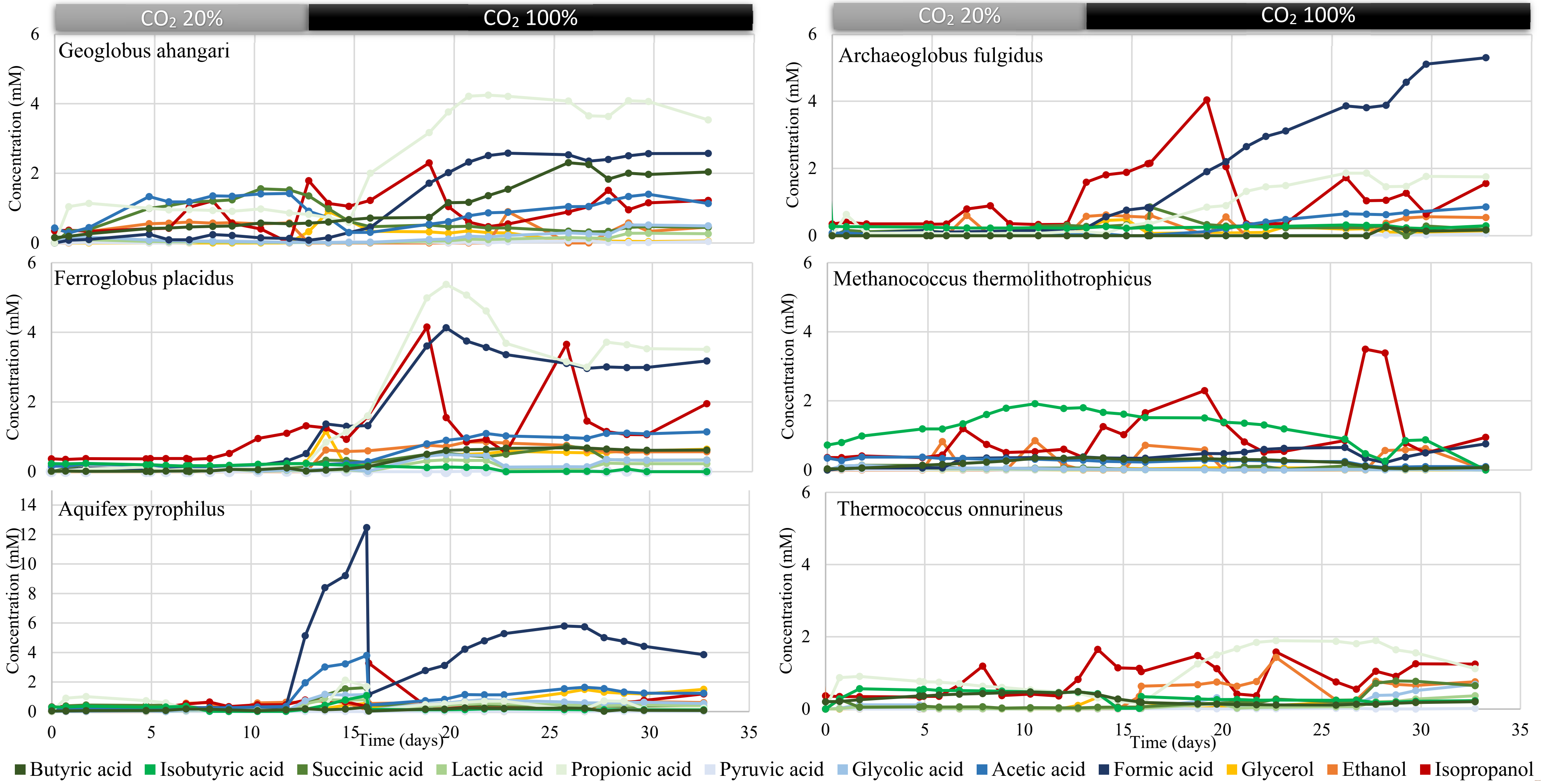

Figure S2. Production of organics in mM during long-term experiments over time. Lactic acid and succinic acid concentrations were ignored for strains supplied with thiosulfate as electron acceptor, as these compounds peak at the same time during HPLC. CO_2 was increased from 20% to 100% on day 13. In *Aquifex pyrophilus*, the media was renewed on day 16 due to high pH.

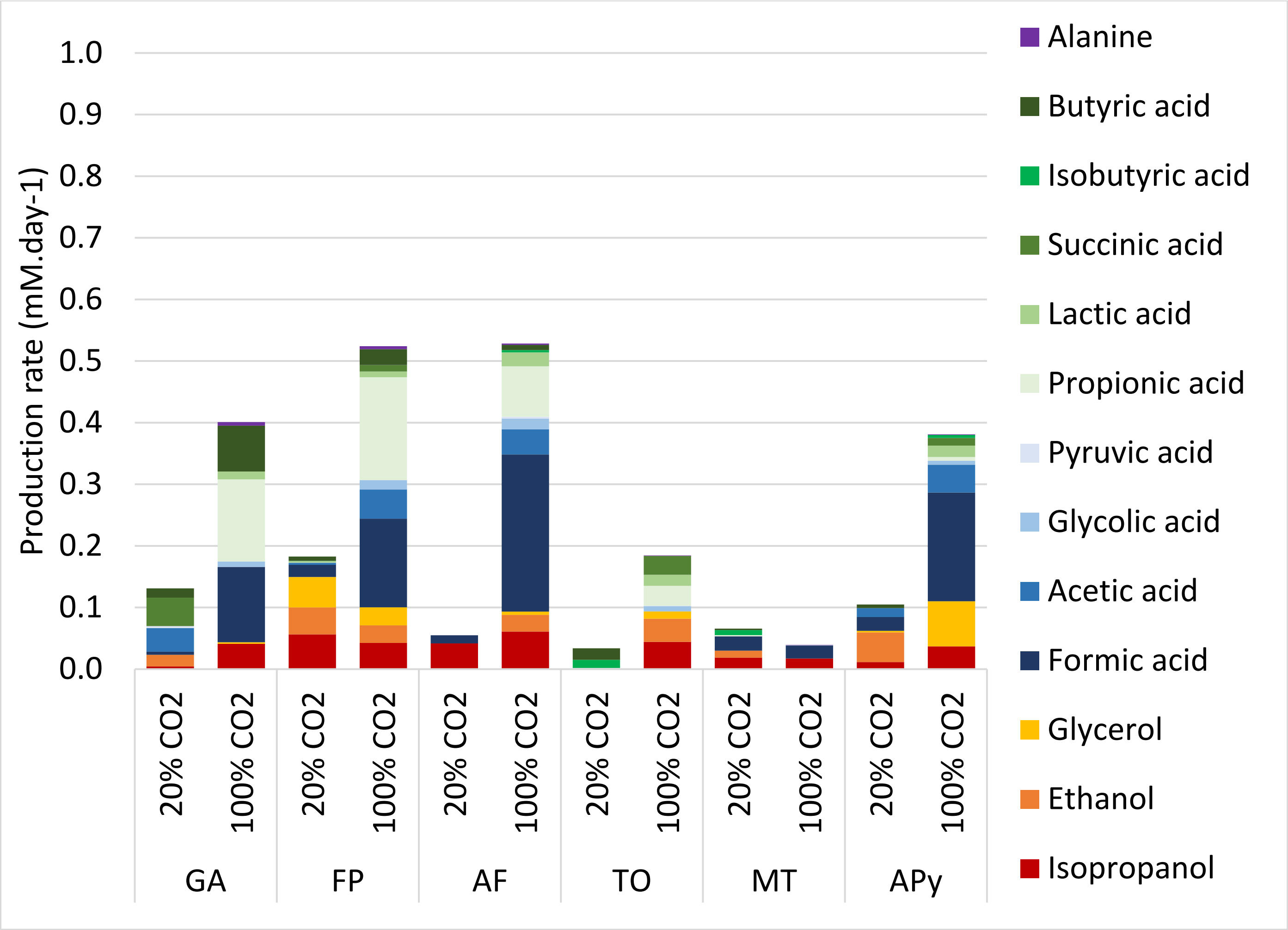

Figure S3. Organic production per day measured by HPLC, NMR, and GC-MS during the 33 days of experiment with the 6 selected strains. This graph show that the increase in organic production is not related to longer cultivation period at 100% CO_2 than at 20% CO_2.

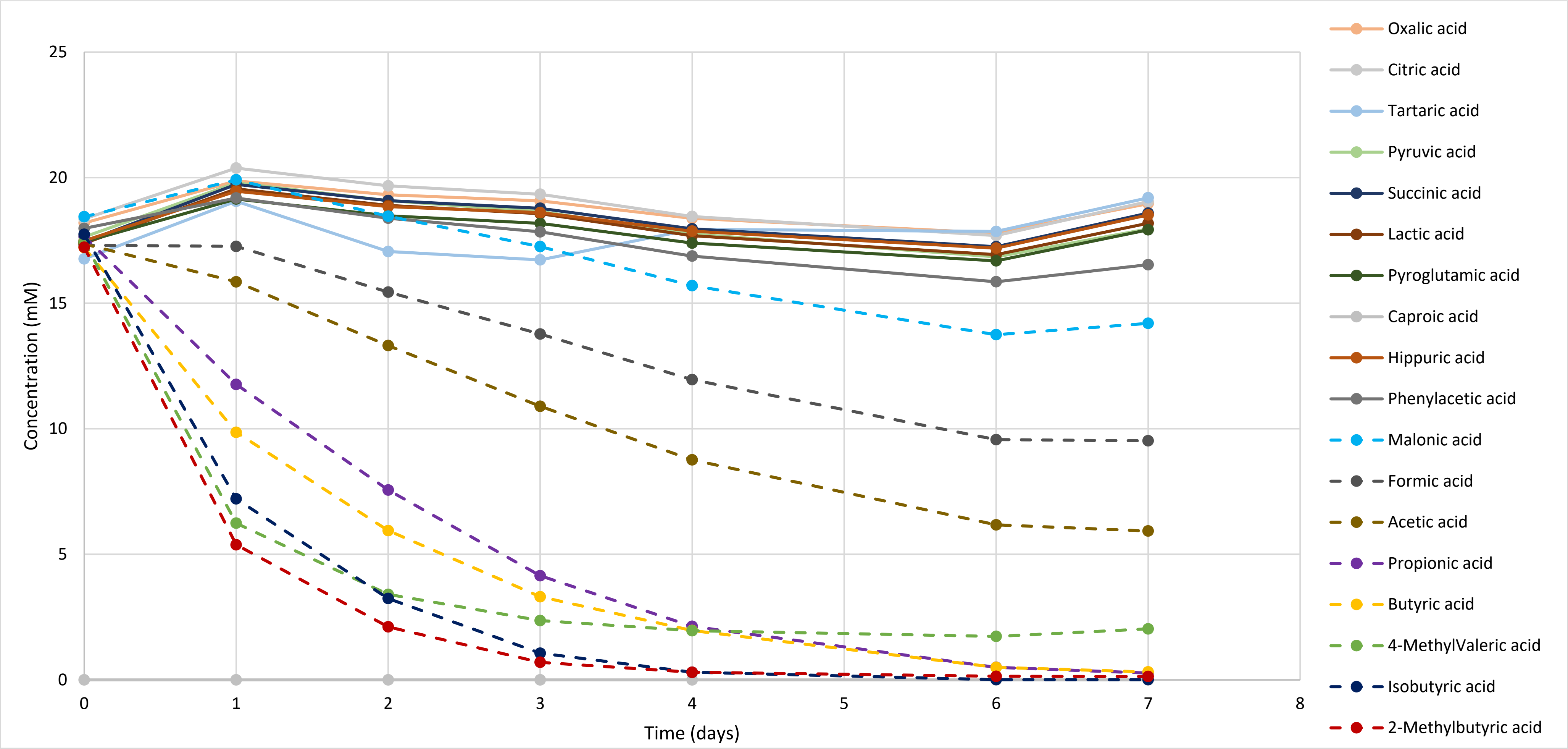

Figure S4. Evaluation of evaporation rates of organic acids in the H-cells over time. The evaluation of evaporation or thermal degradation of organic acids was determined in a non-polarized control in the exact same conditions as the biotic experiments with a mix of each organic acid at 20mM initial concentration and measured each day by HPLC method.

Table S1. Correspondence between codes and enzymes in Figure 5. The presence of gene was assessed from the literature ([21,29]), on the KEGG Database for each strain, or for closest relative strains when not available. X: gene or function not retrieved in the literature. MT*: data from *Methanococcus maripaludis.* Apy*: data from *Aquifex aeolicus.*

Code	Enzyme	GA	FP	AF	TO	MT*	Apy*
**Electron transfer mechanism**							
CYT	Cytochrome	Yes	Yes	Yes	X	X	Yes
HYD	Hydrogenase	Yes	Yes	Yes	Yes	Yes	Yes
**Reverse TCA cycle / TCA cycle**							
ACL	ATP citrate lyase	Yes	Yes	Yes	X	X	Yes
ANH	Aconitate hydratase	X	X	X	X	X	Yes
CS	Citrate lyase	Yes	Yes	Yes	X	X	Yes
FH	Fumarase	Yes	Yes	Yes	X	Yes	Yes
IDH	Isocitrate dehydrogenase	Yes	Yes	Yes	X	Yes	Yes
MDH	malate dehydrogenase	Yes	Yes	Yes	X	Yes	Yes
OFO	2-oxoglutarate:ferredoxin oxidoreductase	Yes	Yes	Yes	Yes	Yes	Yes
SCS	Succinyl-CoA synthetase	Yes	Yes	Yes	Yes	Yes	Yes
SDH	Succinate dehydrogenase	Yes	Yes	Yes	X	Yes	Yes
**Reductive Acetyl-CoA pathway**							
ACS	acetyl-CoA synthase	Yes	Yes	Yes	Yes	Yes	X
CODH	carbon monoxide dehydrogenase	Yes	Yes	Yes	Yes	Yes	X
FDH	formate dehydrogenase	Yes	Yes	Yes	Yes	Yes	Yes
FTS	formyl-THF synthase	Yes	Yes	Yes	X	Yes	X
MTC	methenyl-THF cyclohydrolase	Yes	Yes	Yes	X	Yes	Yes
MTD	methylene-THF dehydrogenase	Yes	Yes	Yes	X	Yes	Yes
MTR	methylene-THF reductase	Yes	Yes	Yes	X	Yes	Yes
**Electrofermentation**							
PFL	pyruvate-formate lyase	X	X	X	Yes	X	X
**Alanine synthesis**							
AST	alanine-synthesizing transaminase	X	X	X	Yes	X	X
**Glycerol synthesis**							
ENL	Enolase	Yes	Yes	Yes	Yes	Yes	Yes
GAPD	phosphoglycerate kinase	Yes	Yes	Yes	Yes	Yes	Yes
GDH	glycerol dehydrogenase	X	X	X	X	X	X
GK	glycerol kinase	X	?	?	Yes	?	Yes
PEPS	phosphoenolpyruvate synthetases	Yes	Yes	Yes	Yes	Yes	Yes
PGM	phosphoglycerate mutase	Yes	Yes	Yes	Yes	Yes	Yes
TIM	triosephosphate isomerase	Yes	Yes	Yes	Yes	Yes	Yes
**Butyrate and Isobutyrate synthesis**							
ALDH	aldehyde dehydrogenase	X	X	X	X	X	X
ALS	acetolactate synthase	Yes	Yes	Yes	Yes	Yes	Yes
ATO	acetyl-CoA C-acetyltransferase	Yes	Yes	Yes	Yes	Yes	X
BCD	butyryl-CoA dehydrogenase	X	X	X	X	X	X
BUK	butyrate kinase	X	X	X	X	X	X
CRT	crotonase	Yes	Yes	Yes	X	X	X
HBD	hydroxybutyryl-CoA dehydrogenase	Yes	Yes	Yes	X	X	X

ICM	isobutyryl-CoA mutase	Yes	Yes	Yes	X	X	X
ILVC	ketol-acid reductoisomerase	Yes	Yes	Yes	X	Yes	Yes
ILVD	ketoisovalerate decarboxylase	Yes	Yes	Yes	X	Yes	Yes
KIVD	alpha-ketoisovalerate decarboxylase	X	X	X	X	X	X
**Propionate synthesis**							
MCD	methylmalonyl-CoA decarboxylase	X	X	X	X	X	X
MME	methylmalonyl-CoA epimerase	Yes	Yes	Yes	Yes	X	X
MMM	methylmalonyl-CoA mutase	Yes	Yes	Yes	Yes	X	X
**Acetate synthesis**							
ACK	acetate kinase	Yes	Yes	Yes	Yes	Yes	X
PTA	phosphate acetyltransferase	Yes	Yes	Yes	X	Yes	X
**Lactate synthesis**							
LDH	D-lactate dehydrogenase	X	X	Yes	X	X	X
**Isopropanol synthesis**							
AACS	Acetoacetyl-CoA synthetase	X	X	X	X	X	X
ADC	acetoacetate decarboxylase	Yes	X	Yes	X	X	X
IDH	isopropanol dehydrogenase	X	X	X	X	X	X
**Ethanol synthesis**							
ADHE	aldehyde-alcohol dehydrogenase	X	X	X	X	Yes	Yes
**Pyruvate synthesis**							
PFOR	pyruvate ferredoxin oxidoreductase	Yes	Yes	Yes	Yes	Yes	Yes
**Glycolate synthesis**							
ACEA	Isocitrate lyase	X	X	X	X	X	X
GHRA	Glyoxylate/hydroxypyruvate reductase A	X	X	X	Yes	Yes	X
**Respiration**							
ETC	Electron transfer Chain	Yes	Yes	Yes	Yes	Yes	Yes
ATPS	ATP synthase	Yes	Yes	Yes	Yes	Yes	Yes
FeR	Iron reducing complex	Yes	Yes	X	X	X	X
DSR	Dissimilatory sulfite reductase	Yes	Yes	Yes	Yes	X	Yes
APR	Adenylyl-sulfate reductase	Yes	Yes	Yes	Yes	X	Yes
SAT	Sulfate adenylyltransferase	Yes	Yes	Yes	Yes	X	Yes
NOS	Nitrous oxide reductase	Yes	Yes	X	X	X	Yes
NOR	Nitric oxide reductase	Yes	Yes	X	X	X	Yes
NIR	Nitrite reductase	Yes	Yes	X	X	X	Yes
NAR	Nitrate reductase	Yes	Yes	X	X	X	Yes

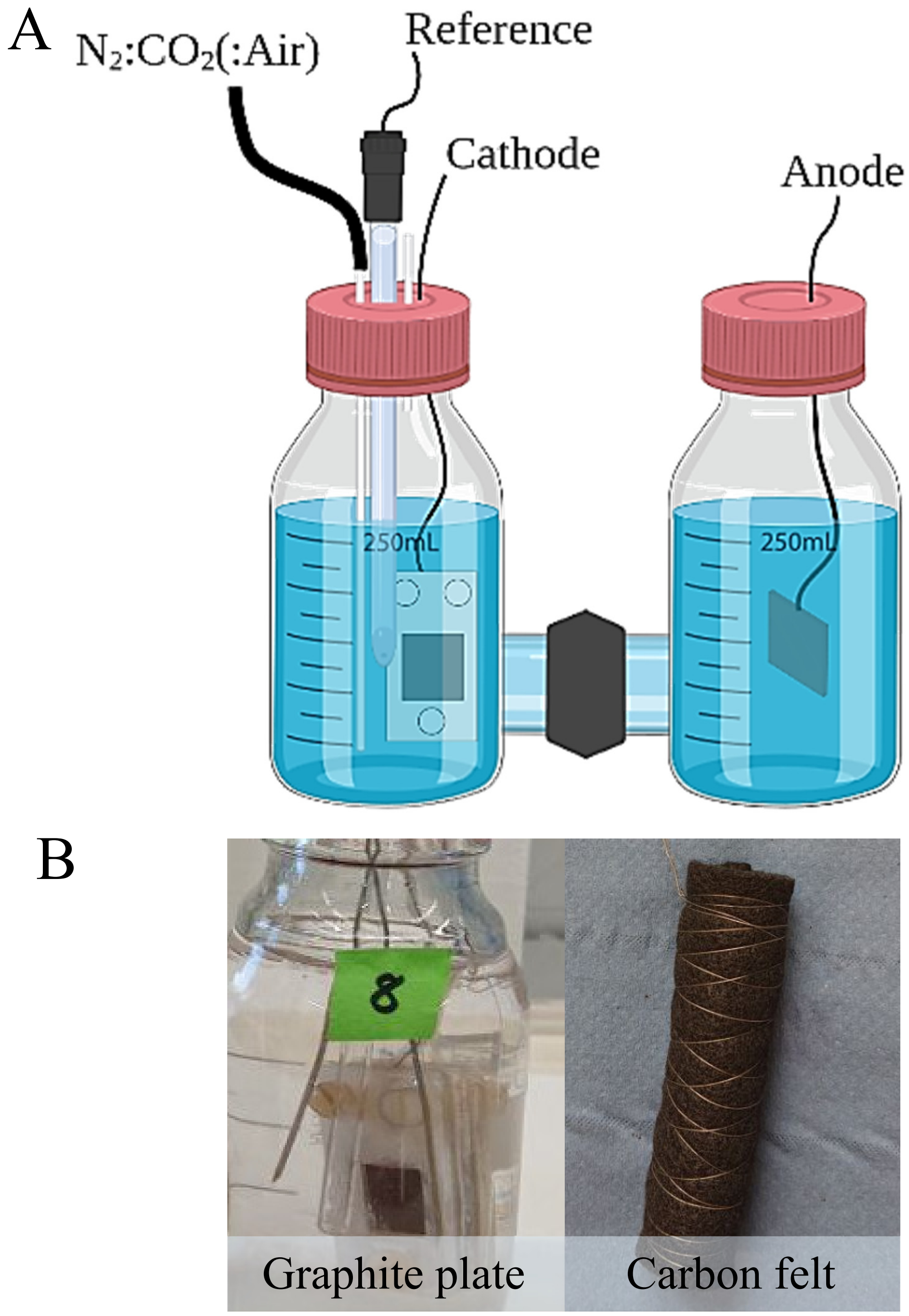

Figure S5. Schematic of the electrochemical system (A) and the 2 types of electrodes used in this study.
